# Locking compression plates versus locking plates for tibial plateau levelling osteotomy in dogs: progression of osteoarthritis, bone healing score and lameness degree

**DOI:** 10.1186/s12917-021-02899-6

**Published:** 2021-05-13

**Authors:** Francesco Macrì, Luca Cicero, Vito Angileri, Vito Biondi, Piero Miele, Lorenzo Scaletta, Giovanna Lucrezia Costa, Giovanni Cassata, Simona Di Pietro

**Affiliations:** 1 Department of Veterinary Sciences, University of Messina, Viale Palatucci s.n.c, 98168 Messina, Italy; 2 Istituto Zooprofilattico Sperimentale della Sicilia “A. Mirri”, Via Gino Marinuzzi 3, 90100 Palermo, Italy; 3 Contrada Torrelunga Puleo, 29, 91025 Marsala, TP Italy; 4 Veterinaria Enterprise Stp S.R.L, via Galvani 33d, 00153 Rome, Italy

**Keywords:** Dog, Locking compression system, Locking system, Tibial plateau levelling osteotomy

## Abstract

**Background:**

The partial or complete cranial cruciate ligament rupture is a common skeletal disease affecting the stifle joint in dogs. The tibial plateau levelling osteotomy, performed with several synthesis systems, changed the approach to its treatment in dogs. The aim of this study was to compare two types of fixation implants, locking compression system and locking system, evaluating radiographically the progression of osteoarthritis of the stifle joint in dogs with complete cranial cruciate ligament deficiency treated surgically with tibial plateau levelling osteotomy. Moreover, we evaluated bone healing and lameness scores to show biomechanical effects by the implant used. Twenty-eight dogs, who met the inclusion criteria, were divided into two groups. Group A: 14 dogs treated using locking compression plates; Group B: 14 dogs treated using locking plates. Radiographic osteoarthritis scores were evaluated up to 1 year following tibial plateau levelling osteotomy. At each visit, animals were clinically and radiographically assessed. Each dog was evaluated before (T0) and after two (T2) and twelve (T12) months after the surgery. At T2 the stage of bone healing was evaluated. The clinical follow up was performed before the surgery and at 10, 15 and 20 days after the surgery, grading the lameness at walk and trot.

**Results:**

An increase in osteoarthritis score at T12 versus T0 in both groups was detected. A decrease of the lameness score was observed in Group A versus Group B. The healing score system at T2 showed a lower score in Group A versus Group B.

**Conclusions:**

The osteoarthritis score following tibial plateau levelling osteotomy did not differ when comparing the two different fixation systems. The locking compression system allowed a more rapid functional recovery of the limb and a quicker bone healing than the locking system. Locking compression system should be carefully considered for dogs subjected to tibial plateau levelling osteotomy surgery, because it may reduce the recovery time.

## Background

The partial or complete Cranial Cruciate Ligament (CCL) rupture is a common skeletal disease affecting the stifle joint in dogs, that can occur suddenly or a consequence of a progressive failure, and results in partial to complete joint stifle instability [1–5].

The cruciate rupture could be correlated with morphological changes in the joint, such as synovitis, articular cartilage degeneration, periarticular osteophyte development and capsular fibrosis that would cause osteoarthritis (OA) [3, 6–9].

In veterinary medicine, radiography is one of the most useful diagnostic tools in the evaluation of joint degeneration and indirect signs of cruciate rupture [6, 10, 11].

Radiographic signs of OA include: osteophytosis, enthesiophytosis, subchondral sclerosis, joint effusion, soft tissue swelling, intra-articular mineralisation, reduction of joint, space and cysts-like lesion [11, 12].

Different scoring systems for the assessment of OA changes have been proposed [8, 13–19].

Clinically, the partial or complete CCL rupture determine a variable degree of lameness that is a common clinical rating system for evaluation of pain in orthopedic patients [20].

The tibial plateau levelling osteotomy (TPLO) changed the approach to treatment of the CCL ruptures in dogs, modifying the biomechanical forces within the stifle by rotating the tibial plateau [21, 22].

Several synthesis systems have been reported to stabilize the osteotomy of the tibia during the TPLO in dogs, assessing also the efficacy and complication rate of the various fixation techniques, through the use of different type of plates, as nonlocking, locking or double locking plates [23], string of pearls locking plate [24, 25], Slocum TPLO plate [26] contoured locking compression plates [27].

Locking compression plates and locking plates rely on completely different mechanical principles to provide fracture fixation: locking compression plates promote a primary bone healing by providing absolute stability; locking plates promote a secondary bone healing through endchondral ossification, functioning as internal fixators in fractures [18].

The aim of this study was to compare two types of fixation implants, locking compression system and locking system, evaluating radiographically the progression of osteoarthritis of the stifle joint in dogs with CCL deficiency treated surgically with TPLO. We hypothesized that the type of fixation system could affect the progression of osteoarthritis over time. Moreover, we evaluated bone healing and lameness scores to show biomechanical effects by the implant used.

## Results

The mean +/− SD and range of age and body weight of dogs was 94.28 ± 25.7 (48–144) months and 30.85 ± 8,87 (20–42) Kg in Group A, and 95.14 ± 29.94 (60–144) months and 29 ± 7,46 (22–42) Kg in Group B, respectively.

In Group A, 65.57% of dogs were females and 34.43% males, while in Group B, 50% were females and 50% males.

In Group A, 57% of right and 42.86% of left pelvic limbs and in Group B 42.85% of right and 57.15% of left pelvic limbs were involved in the injury, respectively.

There were not differences between groups based on age (*p* = 0.603) and body weight (*p* = 0.910).

In Group A, pre and post TPA (degree) were 28.3 ± 1.44 and 6.2 ± 0.8, respectively; in Group B pre and post TPA were 28.2 ± 1.2 and 5.8 ± 0.4, respectively.

The Kendall test showed a high level of inter-observer concordance (W = 1) in both groups for OA, bone healing and lameness scores.

The statistical analysis showed that OA score increases in both groups 12 months after surgery (*p* = 0.001), while there was no difference between groups within a single time point (T0: *p* = 1.000; T12: *p* = 0.702). Instead, a significant reduction of inflammation score (infrapatellar fat pad score) was observed over time within each group, while no differences were observed between groups at T0 and T12, with *p* = 0.352 and *p* = 0.769, respectively (Table 1).

**Table 1:** Median and range of OA score and inflammation score in the Group A and B

Experimental group	***OA score***		***Inflammation score***	
	***T0***	***T12***	***T0***	***T12***
**A**	15 (6–20)	21 (13–28)*	2 (2–2)	1 (1–1)*
**B**	14 (9–18)	19 (14–28)*	2 (1–3)	1 (0–1)*

*OA* Osteoarthritis, *T0 and T12* Data points investigated; *vs T0 *p* ≤ 0.001 (statistical difference)

**Fig. 1:**
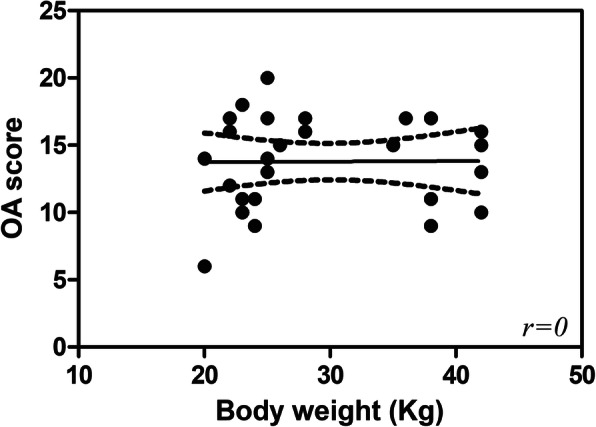
Scatter plot illustrating no correlation between subject’s body weight at presentation and osteoarthritis (OA) score (*r* = 0)

No correlation was noted between subject’s body weight and OA score with *r* = 0 (Fig. 1).

**Fig. 2:**
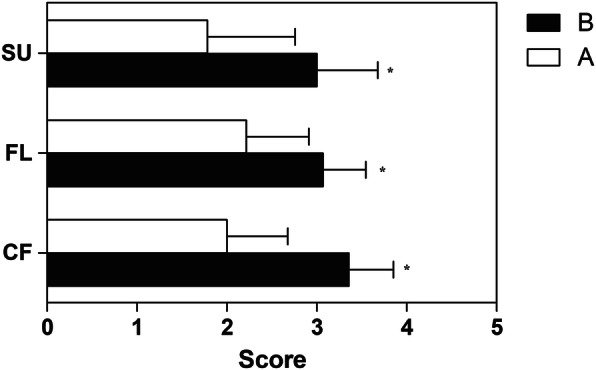
The healing score system of the experimental groups, for three considered variables: callus formation (CF), fracture line (FL), union stage (US). Statistical comparison (*vs Group A *p* ≤ 0.01) in time point T2, 2 months after surgery; the bar represents the mean with standard deviation

The healing score system, evaluated 2 months after surgery (T2), showed a lower score in Group A compared to Group B, for the three considered variables: callus formation, fracture line and stage of union (*p* = 0.000, *p* = 0.001, *p* = 0.002, respectively) (Fig. 2).

**Fig. 3:**
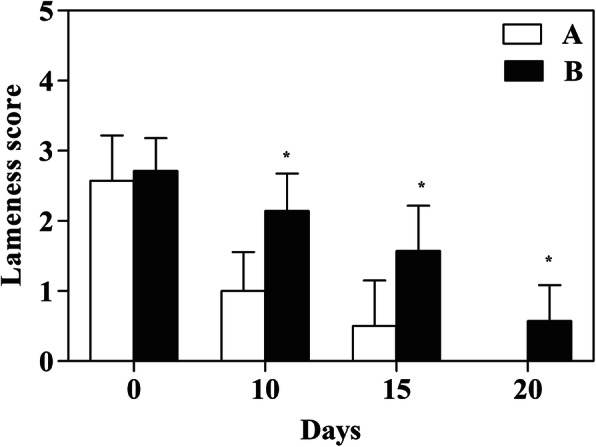
The clinical lameness score of the experimental groups. Comparison of the values for lameness score in the locking compression plate and locking plate treated groups over the 20-days evaluation after surgery. Statistical differences (* vs Group A *p* ≤ 0.01) in specific time points, and the bar represents the mean with standard deviation

The lameness degree before the surgery (T0) showed any differences between groups (*p* = 0.701), while along the time line a reduction of the lameness score in both groups was observed. The comparison between groups at the three postoperative time points (T10, T15 and T20) showed a significant difference, with a lameness score in Group A lower than in Group B (*p* = 0.000, *p* = 0.001, *p* = 0.009, respectively) (Fig. 3).

## Discussion

Results of the present study showed a similar trend of osteoarthritis scores in both fixation methods: OA score increases significantly in both groups 12 months after surgery.

The statistical analysis showed a reduction of synovial volume in the stifle after the surgery in both groups, as showed through the radiographic changes in the shape of the intrapatellar fat pad. This finding seems to confirm the efficacy of both fixation systems.

Results of the present study show a significant difference between groups for the healing score system (considering the callus formation, fracture line and stage of union), with a lower score in Group A than in Group B.

A possible explanation to this finding is that locking plates and locking compression plates rely on completely different mechanical principles of bone healing and so provide for a different biologic environment for bone union. The interfragmentary compression and absolute stability, provided by locking compression plates (Group A), determine a primary bone healing with early obliterated fracture line and early achieved stage of union. In Group B, locking plates act as internal fixators in fractures with a wider gap and interfragmentary movements, determining a secondary bone healing with trabecular bone formation [18, 28].

From the obtained results, it would appear that compression plate might reduce the osteotomy consolidation time, resulting in faster healing. Further studies are needed to deepen this topic.

Moreover, we believe that the absolute stability provided by the compression plate (rigid fixation) have also contribute to the reduction of the lameness as early as 20 days post-operative.

In our study, due to the difficulty in obtaining a radiographic caudo-cranial projection of the stifle joint 1 year after surgery (due to the reticence of owners in sedating dogs), we used a modified DeLuke and colleague’s scoring system, with grade ranging from 0 (no changes) to 3 (severe changes), using only 14 radiographic points without the execution of orthogonal projections in follow up visits. De Bruin et al. [29] evaluated the progression of the osteoarthrosis in contralateral canine stifle joint of dogs with a CCL rupture using only the medio-lateral projection and applying a simple score with ten points.

In the present study the mediolateral view of the stifle was used for evaluate the osteophytic changes in patients. The presence of osteophytes is an important sign of joint instability and the score of these is closely correlated with the progression of the disease [9]. Furthermore, the assessment of osteophytes seems to have several advantages. They occur early in the pathological process, being radiographically visible at the margins of the femoral trochlea already 2 weeks after the cruciate ligament rupture and some Authors report their formation already 3 days after the trauma [10, 30].

It is unknown if there are any incidence in dogs for the development of osteoarthritis secondary to other factors such as breed, age, sex and obesity [31]. Some Authors reported that there is no significant correlation between the animal’s body weight and the progression of osteophytes [17]. In contrast, other studies demonstrated that a greater body weights could be a predisposing factor of the osteoarthritis and disease progression in dogs [32].

In the present study, no significant correlation was observed between dog’s weight at presentation and OA score, according to Rayward et al. [17]. The exclusion of dogs weighing less than 20 kg from our study may explain the discrepancy with the results of Gilbert et al. [33], who reported that dogs weighing > 35 kg had a higher total osteoarthrosis score than smaller dogs. In this study a breed predisposition to the development of osteoarthritis has not been assessed due to the reduced number of pure-breed dogs (*n* = 9/28) versus the cross-breed dogs (*n* = 19/28) in the study.

The study presents some limits. The first is the small number of dogs enrolled and future studies should include more cases of TPLO using both implants, also in various breeds. Moreover, during the follow-up it was not possible perform the functional evaluation of stifle joint and TPA measurement due to the reticence of owners in sedating dogs. It would be interesting to evaluate the correlation between 1-year TPA, osteoarthritis progression and type of implant used.

In conclusion, the findings of this study confirm that the progression of radiographic signs of osteoarthritis occurred in all dogs with cruciate rupture after TPLO surgery despite the fixation system used. The author suggest that the locking compression plates would be the most suitable choice because it may reduce the recovery time. Obviously, the final surgical choice should be depending on the experience of the operator and owner compliance.

## Methods

### Case selection

The study was conducted in compliance with the guidelines outlined in the US Animal Welfare Act and in the European Directive 2010/63/EU for animal experiments and the Good Clinical Practice (Federation of Veterinarians of Europe, FVE, Code of Good Veterinary Practice, 2003).

Forty dogs were diagnosed with complete monolateral cranial cruciate ligament rupture and were enrolled in this study after the consent provided by owners. The cruciate rupture was clinically diagnosed by a history of hind limb lameness found during an orthopaedic examination (a positive cranial drawer and/or positive cranial tibial thrust), by the presence of joint effusion or knee osteoarthritis on radiographs and by the laboratory analysis of the synovial fluid. Dogs with evidence of non-orthopedic systemic or unrelated orthopedic diseases and with a bodyweight less than 20 kg were excluded. Dogs were also excluded if there is an arthroscopy evidence of meniscal injuries (tears, compression and folded caudal horn), to eliminate the different post operative outcome associated to these variables, and if the radiographs obtained immediate postoperatively showed a tibial plate angle (TPA) superior than 7 degree, because the smaller angles may represent the optimal angle of tibial plateau rotation providing joint stability in cranial cruciate ligament-deficient stifles [34].

Twenty-eight dogs who met the inclusion criteria were selected among all animals (Table 2).

**Table 2:** Summary of dog signalment and involved limb

Case	Breed	Sex	Age (months)	Body weight (Kg)	Involved limb
1	Cross-breed	F	96	36	Right
2	Cross-breed	F	120	24	Right
3	Cross-breed	F	48	23	Left
4	Cross-breed	M	48	22	Right
5	Golden Retriever	F	96	38	Left
6	Cross-breed	F	96	28	Right
7	German Sheperd	M	96	42	Right
8	Cross-breed	F	144	22	Right
9	Cross-breed	F	108	42	Right
10	Labrador	M	72	25	Left
11	Cross-breed	M	108	42	Left
12	Cross-breed	F	108	42	Left
13	Cross-breed	M	84	26	Right
14	Border collie	F	96	20	Left
15	Cross-breed	F	96	38	Left
16	Cross-breed	M	120	24	Left
17	Beagle	M	132	28	Right
18	Cross-breed	F	48	23	Right
19	Cross-breed	F	144	22	Left
20	Golden Retriever	F	96	35	Left
21	Cross-breed	M	108	42	Left
22	Cross-breed	M	84	20	Right
23	Cross-breed	F	60	23	Left
24	Labrador	M	72	25	Right
25	Boxer	F	60	25	Left
26	Boxer	F	25	25	Right
27	Cross-breed	M	38	38	Left
28	Cross-breed	M	38	38	Right

### Preoperative planning

Preoperative orthogonal radiographic views were obtained. The mediolateral view was used for preoperative measurements to plan tibial osteotomy and to assign a radiographic OA score (T0). The caudocranial radiographic projection was used for preoperative measurements to plan the length of screws. The TPA was measured on a mediolateral radiograph centred on the stifle joint including also the tarsus; the tibia was parallel to the X-ray film cassette, as reported by Reif et al. [35].

### Surgical procedure

Dogs enrolled in this study underwent to TPLO surgical technique combined with artroscopy to exclude concurrent meniscal tears. Two different plate systems were used: locking compression plates (TPLO Clover plate Fixin®: group A; *n* = 14) and locking plates (TPLO Locked plate Fixin®: group B; *n* = 14). Subjects were randomly divided in the two groups.

The dogs were premedicated with 0.04 mg kg^− 1^ of methadone hydrochloride (Semfortan®, Eurovet Animal Health, Netherland) and 0.03 mg kg^− 1^ of acepromazine (Prequillan®, Fatro SpA, Italy) given intramuscularly. Anaesthetic induction was achieved with 4 mg kg^− 1^ of propofol (Rapinovet®, Intervet, Italy) given intravenously and maintained with isoflurane in oxygen. Epidural lidocaine (Lidocaina 2%, Zoetis, Italy; 6 mg kg^− 1^) and subcutaneously cefovecin (Convenia®, Zoetis, Italy; 8 mg kg^− 1^) was administered only once. The surgical team (F.M., P.M., L.S., and V.A.) performed the TPLO surgery.

The surgical procedure was performed without an alignment jig. A medial surgical approach to the proximal tibia was used. Two distances (D1 and D2) were measured to determine the intraoperative landmarks and the positions were marked using electrocautery. D1 is the distance from the insertion of the patellar tendon to the osteotomy perpendicular to the cranial border of the tibia. D2 is the distance between the insertion of the patellar tendon and the exit of the cranioproximal osteotomy from the tibia.

The size of a TPLO saw, used for the osteotomy, had been predetermined from the preoperative radiographs. After the osteotomy, a Steinmann pin (2.0 mm or 2.5 mm in diameter) was used to rotate the proximal segment until the distance required to create a postoperative TPA of 5 degrees. Next, a Kirschner wire (1.0 mm or 1.25 mm in diameter) was inserted through the proximal part of the tibial tuberosity to the proximal bone segment for temporary fixation.

The proximal bone segment was stabilized with a predetermined TPLO plate, locking head screws, and cortical screws. After the plate setting, Steinman pin and Kirschner wire were removed.

### Postoperative assessment

The immediate postoperative radiographic study was performed under general anaesthesia, included a mediolateral and caudocranial projection of the stifle, as standard projections used for CCL injuries. The unaffected leg was pulled away (abduct or pulled cranially). After, a large film cassette was placed under the patient. The angles in between joints (femorotibial and tibiotarsal) was 90° and in radiographic pictures the two condyles was perfectly overlapped. The caudocranial projection was performed with the dog placed in the sternal recumbency, with the affected limb completely extended in a caudal direction and straight so that the patella remained on the cassette. The patella was centred between both fabellae. The medial border of the calcaneus was aligned with the centre of the distal tibia. The X-ray beam was centred at the level of the middle portion of the tibial diaphysis (included the stile, entire tibia and tarsus in the exposure). The pes was not twisted or rotated.

The postoperative TPA was measured on these radiographs, and the osteotomy site, apposition, implant position, and limb alignment were evaluated.

All dogs underwent the following postoperative protocol: restricted activity (no running, no jumping, no free access to stairs, no active play) for the next 6 weeks designed to complete healing of the surgical site; keeping the incision clean and dry and no licking; monitoring the surgical incision daily to detect signs of infection.

Each dog was subjected to two radiographic follows up studies at two (T2) and twelve (T12) months after surgery, performing a medio-lateral view with dogs awake.

Radiographs were reviewed by three veterinary radiologist (SD, LS, PM) working independently, blinded to breed, age and weight of the patients; the radiologist were unaware of the type of fixation used because each plate was digitally covered.

At T0 and T12, OA score was determined in the medio-lateral view of the 28 joints affected, using a modified 14 assessment point OA scale (Table 3) to grade features associated with OA, assigning a score from 0 to 3 for each point [15]. A score of 0 indicates no signs of apparent degeneration, 1 mild arthritic changes, 2 moderate arthritic changes and 3 severe arthritic changes; the final score could have been in between 0 and 42.

**Table 3:** Fourteen-point scale used to grade the features of osteoarthritis and related radiographic signs

Peri-articular osteophytes, femoral condyles	Femoral subtrochlear lysis
Peri-articular osteophytes, proximal tibia	Osteophytes, tibial plate
Apical patellar osteophytes	Basilar patellar osteophytes
Synovial volume (changes in the shape of the intrapatellar fat pad)	Peri-articular osteophytes, femoral trochlea
Distal femoral condylar remodeling	Peri-articular osteophytes, fabellae, lateral and medial gastrocnemius and popliteal sesamoids
Cranial apical patellar entesiopathy	Periarticular osteophytes, cranio-proximal tibial
Periarticular osteophytes, caudo-proximal tibial	Tibial condylar region remodelling

In particular, among the radiographic signs to grade the pattern of OA, the presence of acute inflammation was indirectly inferred when a soft tissue opacity was present cranial to the joint space (infrapatellar fat pad) on animals with acute signs of lameness and no evidence of periarticular bone formation.

At T2, each osteotomy was evaluated for the stage of bone healing following a radiographic grading system by Hammer et al. [36] reported from Boero Baroncelli [37], which evaluate the callus formation, fracture line and stage of union, with a score from 1 (homogenous bone structure; obliterated fracture line; achieved union) to 5 (no callus formation; distinct fracture line; not achieved union).

The clinical follow up evaluations was performed before the surgery and at 10, 15 and 20 days after the surgery, grading the lameness at a walk and a trot, with a scoring from 0 to 5: 0 walks or trots normally; 1 slight lameness; 2 obvious weight-bearing lameness; 3 severe weight-bearing lameness; 4 intermittent non- weight-bearing lameness; 5 continuous non-weight-bearing lameness, as reported by Fossum [38].

An independent investigator, unaware of the fixation system used, assessed the lameness.

### Statistical analysis

Nonparametric statistical analysis was performed using the statistical software SPSS 15.0 (IBM Company, Italy). The quantitative variables, as age (months) and weight (Kg) of dogs, and pre and post TPA degree were expressed as the mean +/− SD and range. The frequency distribution of gender and affected limb were determined. Kendall’s coefficient of concordance (W) was used to measure agreement among observers; the scores were expressed as the median and range. The Wilcoxon test was performed to assess changes in OA grade into each group at the different time points and the Mann-Whitney U test was performed to evaluate the differences between groups. The Pearson’s correlation between the body weight of subjects and the OA score was also performed at T0. The Mann-Whitney U test was also applied in order to compare the postoperative bone healing between groups. Intra and inter-groups variation in lameness grade was assessed by the same above-mentioned tests. For all statistical tests, significance was set at *p* < 0.05.

## Data Availability

The datasets used and/or analyzed during the current study are available from the corresponding author on reasonable request.

## Abbreviations

CCL: Cranial cruciate ligament
OA: Osteoarthritis
TPLO: Tibial plateau levelling osteotomy
